# Regional impact of COVID-19 on the production and food security of common bean smallholder farmers in Sub-Saharan Africa: Implication for SDG's

**DOI:** 10.1016/j.gfs.2021.100524

**Published:** 2021-06

**Authors:** Eileen Bogweh Nchanji, Cosmas Kweyu Lutomia

**Affiliations:** a International Center for Tropical Agriculture, Nairobi, Kenya; b Kenya Agricultural and Livestock Research Organization, Nairobi, Kenya

**Keywords:** COVID 19, Food security, Agricultural production, Sub-saharan Africa, SDG'S

## Abstract

Concerns about the implications of COVID-19 on agriculture and food security in Sub-Saharan Africa abound. Containment measures in response to the pandemic have markedly different outcomes depending on the degree of enforcement of the measures and the existing vulnerabilities pre-COVID. In this descriptive study, we document the possible impacts of the pandemic on bean production and food security using data collected from March to April 2020 in eleven countries in four sub-regions in Sub-Saharan Africa. The results reveal that COVID-19 created significant bean production challenges across the sub-regions, including low access to seed, farm inputs, hired labor, and agricultural finance. We also show that COVID-19 threatens to reverse gains made in the achievement of Sustainable Development Goals number 1 and 2. Countries in Southern and Eastern Africa are likely to suffer temporal food shortages than those in Western and Central Africa. Although governments have responded by offering economic stimulus packages, much needs to be done to enable the sub-sector to recover from ruins caused by the pandemic. We recommend building sustainable and resilient food systems through strengthening and enabling public-private partnerships. Governments should invest directly in input supply systems and short food supply chains through digital access and food delivery.

## Highlights

• COVID-19 threatens to reverse gains made in the achievement of Sustainable Development Goals.
• We can only achieve sustainable and resilient food systems through strengthening and enabling public-private partnerships.
• The pandemic has necessitated a much needed holistic discussion around the complex food systems.
• The pandemic’s effect on food consumption patterns triggered changes food quality and quantity negatively.

## 1 Introduction

Sustainable food systems are needed to ensure sustainable food production that meets the growing world population's demand projected to be 9.8 billion people by 2050 (UN, 2017). Essentially, food systems take into account the complex web of production, distribution, and consumption of food. The complex global food chains require well-functioning markets and dynamic linkages for safer and cheaper movement of food from production to consumption (Fan, 2017). However, this has not been the case due to production, post-harvest, marketing, and consumption inefficiencies (Alexander et al., 2017). These inefficiencies jeopardize efforts towards food and nutritional security.

Besides inefficiencies, food systems in developing countries were affected by adverse effects of climate change, pests, and diseases before the pandemic (Pratt, 2015; Shimeles et al., 2018; Pais et al., 2020; GAIN, 2020; Gralak et al., 2020). The rapid and extensive spread of coronavirus in unprecedented ways is already exacerbating the food systems efficiencies. FAO (2020) highlighted multiple ways that the global pandemic would affect access to food, food security, and nutrition. In response to the spread of the deadly virus, countries imposed containment measures with varying degrees of strictness. Depending on the level of strictness, the measures have caused uncertainty in food production, distribution, and consumption, with concerns over its implications on the already dire food security problems in developing countries (Nchanji et al., 2020). Thus, the pandemic has necessitated holistic discussions around the complex food systems.

The pandemic-related food security challenges are expected to slow down progress towards eliminating hunger and poverty in Sub-Saharan Africa. For instance, 55 percent of the world's hungry people and 70 percent of the world's poorest people lived in Africa before COVID-19 (UN, 2017). Thus, COVID-19 is aggravating an already existing food production, distribution, and consumption inefficiencies in the region, negating progress towards eliminating hunger.

Furthermore, Akinwotu (2020) reports that more than twenty-two million people in West Africa needed food assistance before the pandemic, and more than 6 million would be in severe food crisis during the health crisis.

The expected food security problems are attributed to containment measures (Table 1) that have resulted in low incomes, which reduce individual and household food purchasing power (FAO, 2020). Job losses and reduced incomes, and private transfer payments have caused poor households to become more food insecure (Foh et al., 2020). Moreover, border restrictions have reduced food trade, exacerbating food insecurity in food-deficit countries (Foh et al., 2020). For instance, 15 percent of imported food in Kenya before the pandemic was sourced from countries that imposed export restrictions (Foh et al., 2020). Additionally, food production has been negatively affected by disruptions in agro-input markets. Logistics and food markets are also affected, causing food trucking capacity to decline.

**Table 1:** Summary of government COVID-19 containment measures by country.

Country	Policies and containment measures
Kenya	Partial lockdown (May 7), partial border closure (May 16), cessation of movement in high-risk counties/regions (April 6), dusk to dawn curfews (March 29), social distancing and mandatory mask-wearing (April 6), closure of schools (March 15), churches and non-essential businesses (April 11), restricted air transport (8 April), ban on travel across Tanzanian border (16 May) (Un Migration Agency, 2020).
Uganda	Nationwide lockdown (March 31, eased June 2), curfew (March 31), closure of non-food selling businesses (March 31), and restricted transportation (March 31, enhanced April 10), social distancing and mandatory mask-wearing (Anadolu Agency, 2020; Un Migration Agency, 2020)
Tanzania	Closure of schools, ban on public gatherings (17 March), and advise encouraging people to avoid unnecessary movements. No formal internal movement restrictions. Suspension of air travel and intercountry public bus services (March 25, reinforced April 11, relaxed on May 14, and lifted on May 18), Kenya border closure (May 17) (Anadolu Agency, 2020; Kell, 2020).
Madagascar	State of health emergency, suspension of regional and international flights (March 20), total lockdown (March and July 6–20) in Analamanga and Antananarivo, restricted business operation hours, curfews, and restricted transportation services (March–September 2020) (World Aware, 2020a).
Burundi	Flights suspension (20 March), blockage of cargo transportation from EAC member countries (20 March), closure of borders with Rwanda and DR Congo, reopening border to cargo transportation (April 13) (Un Migration Agency, 2020).
Zambia	Shutdown of educational institutions and foreign travel restrictions (March 17), border closure (May 10), partial lockdown, partial closure of non-essential businesses, social gatherings ban, and suspension of cross border passenger and cargo transportation services (Nkomesha, 2020; World Aware, 2020b).
Zimbabwe	Declaration of state of disaster (March 20), prohibition of gatherings and national lockdown and curfews (March 30), and closure of non-essential services and business operations (KPMG, 2020).
Eswatini	Cancellation of public events (March 14), restrictions on internal and international movement (March 17), close of non-essential public transport (March 27) (Ask About)
Lesotho	Closure of borders to air and land travel, ban of public transport, closure of recreational facilities (March–August), partial easing in August (World Aware, 2020c).
Cameroon	International travel control (March 13), closure of schools and public transport, and restriction on gatherings and internal movements (March 18), and workplace closure (May 1) (Ask About, 2020)
Burkina Faso	Closure of borders, airports (March, 20) and nationwide curfew (March 20) (Gongo, 2020)

Adapted from Nchanji et al., 2021a, Nchanji et al., 2021b.

Therefore, this paper identifies the immediate implications of COVID-19 and containment measures on bean farmers’ production and food security in four sub-regions of Sub-Saharan Africa. Common bean is a widely grown leguminous crop in the region, and its importance is reflected in its integration in different cropping systems and multiple benefits ranging from food security, nutrition and health, and soil nutrient recapitalization (Snap et al., 2018). The paper also discusses the implications of COVID-19 disruptions for agricultural production on SDGs.

## 2 Conceptual framework

COVID-19 is expected to impact food security directly and indirectly. The direct impact is linked to farms and food businesses closing due to coronavirus (Devereux et al., 2020). On the other hand, indirect impacts are linked to lockdown, border closures, social distancing, and restricted transportation and movement imposed by governments to minimize the spread of the virus and loss of life. Depending on the level of strictness, government restrictions create economic hardships through reduced earnings and economic activities, leading to food insecurity and hunger. Thus, the direct and indirect effects of the current pandemic can be conceptualized theoretically using FAO's four pillars of food security, the United Nations Committee on World Food Security approach, and Sen's entitlement frameworks as reviewed by Devereux et al. (2020).

FAO's four pillars of food security associate food security with availability, accessibility, utilization, and food stability at micro and macro levels. Food availability is a measure of food supply, while access is having the ability to have food either from own production or markets. Utilization refers to the dietary quality of food. Stability is a dynamic concept that requires that availability, access, and utilization are stable or adequate over time (FAO, 2008). Concerns over cross-border restrictions and logistic challenges created by containment measures are based on the pandemic's indirect impacts on food security's four pillars. For instance, the rationale for border closure and export restrictions as strategies of building national food reserves due to uncertainty could impact availability and access to food in most countries in Sub-Saharan Africa because of their dependence on global trade (Devereux et al., 2020; Foh et al., 2020). In addition, social distancing, pay cuts, job losses, and high food prices due to reduced food imports threaten food security. Closure of informal markets may also disrupt the stability of access and utilization of food among poor consumers who may be unable to afford food from formal outlets.

The food systems approach focuses on all activities and agents involved in food production, processing, distribution, and consumption, including socioeconomic and environmental outcomes (HLPE, 2017). In other words, the possible impacts of the COVID-19 could be viewed from the lenses of the direct and indirect effects on producers, business people, institutions, and consumers, and activities that the agents are involved in. The approach also focuses on dynamic interdependencies and interrelationships among food system players and activities (Ericksen, 2008; Devereux et al., 2020). In other words, the food systems approach recognizes that the effect of coronavirus and containment measures on one node of the food chain is transmitted to several other agents and activities. For instance, restricted mobility may reduce farmers’ access to inputs, labor, and financial services, disrupting food production and distribution. On the other hand, lockdowns and closure of food markets could result in unstable availability and access to food in consumption hubs and food losses and low prices in production hubs due to a glut of perishable food products. These contribute to food systems inefficiencies and possible food insecurity and hunger.

The entitlement approach was envisaged by Amartya Sen to assess food security implications during famines. According to Sen (1981), food security can be viewed from a socioeconomic dimension. The socioeconomic dimension of Sen's proposition is that access to food is uneven and defined by wealth and resources, which are unevenly distributed in developing countries. The approach identifies four sources of food, including own production, own labor, trade, and transfer entitlement. Devereux et al. (2020) observe that the four food sources are impacted by pandemics, such as COVID-19, from an economic and food perspective because of the inherent inequalities.

This paper focuses on bean producers in Sub-Saharan Africa characterized by high-income inequality (UNDP, 2017), whose countries have imposed varying restrictions in response to the spread of the coronavirus (Table 1). This suggests possible negative impacts of COVID-19 on food security situations at country and regional levels. Thus, concepts from the three approaches are applied in contextualizing the impact of COVID-19 on bean production and food security.

Farming is an important economic activity in rural areas in Sub-Saharan Africa. Thus, the direct impacts of COVID-19 could disrupt farming when the virus infects members of farming households and agricultural workers. Second, bean production could be affected indirectly when farmers are unable to access farm inputs and finance due to containment measures. Restrictions of internal and external transportations could constrain access to both formal and informal markets. For households with farming as a secondary or complementary economic activity, COVID-19 could undermine own-labor as a source of food. In other words, rural households who engage in income-earning activities such as wage labor, business, and salaried employment have to contend with lower earnings due to reduced working hours, pay cuts, or job losses. The informal workers are expected to be the worst hit because of the lack of unemployment and social support programs. Additionally, farming households that depend on off-farm income and private transfers from family members and relatives to invest in farming could also be impacted. Besides, remittances have been reduced, and public transfer mechanisms such as school feeding programs are no longer in place. Thus, the pandemic's triple effects are low food production, lack of economic access to food markets, and many mouths to feed. These negatively impact food security in the short term and mid-term.

## 3 Study area and methodology

### 3.1 Study area

**Fig. 1:**
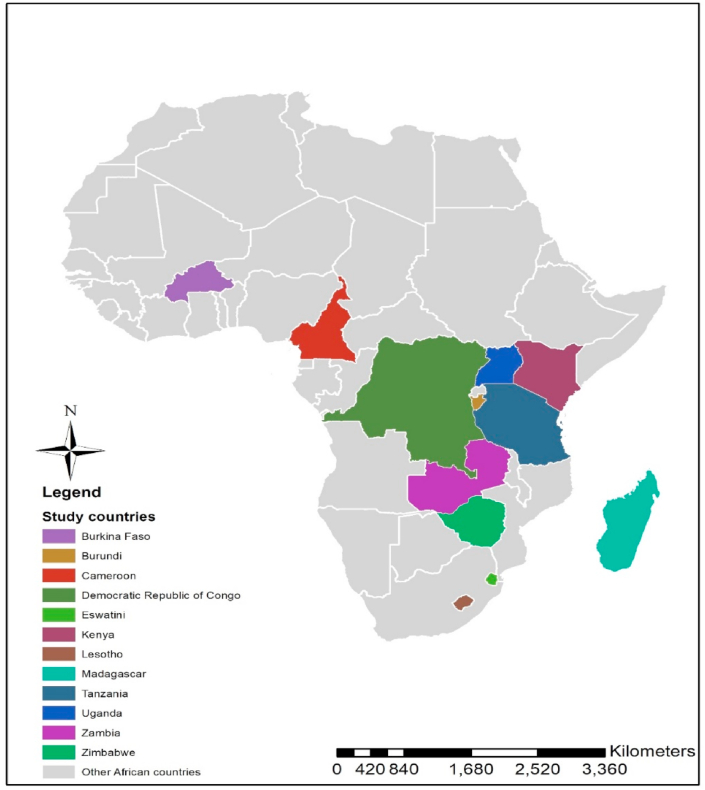
Map of study areas.

The research study was carried out in high bean producing areas of the West & Central Africa Bean Research Network (WECABREN) – Cameroon and Burkina Faso, Southern African Bean Research Network (SABRN) – Eswatini, Lesotho, Zimbabwe and Zambia, and East and Central African Bean Research Network (ECABREN) – Burundi, Kenya, Tanzania, Uganda and Madagascar - supported by the Pan Africa Bean Research Alliance (PABRA) from March–April 2020 as shown in Fig. 1. These are eleven out of the 32 countries supported by the Pan African Bean Research Alliance (PABRA) working on improving food security, income, and health of smallholder farmers and other bean value chain actors like aggregators, consumers, seed companies, traders, processors, researchers, etc. across the bean corridors.

Common bean does not tolerate long periods of frost at any growth stage; it is a warm-season crop. This crop requires only moderate amounts of rainfall, and being a short-season crop; it matures between 65 and 200 days (Wortmann et al., 1998). Information from the second bean atlas shows that beans are mainly grown in the highlands and Mid elevation (1000–1500 m.a.s.l.) in Eastern Africa and Southern Africa and Mid and low elevation (1000 m.a.s.l.) in West and Central Africa and latitudes 6 ^o^S and 13 ^o^N and east of longitude 27 ^o^E (Eastern Africa), South of latitude 6 ^o^S (Southern Africa) and west of longitude 15 ^o^E (West Africa) (Katungi et al., 2020; Wortmann et al., 1998).

### 3.2 Methodology

The data were collected by gender focal person in the National Agricultural Research Systems (NARS), focusing on common beans. Data was collected from 291 bean farmers in rural areas through web-based surveys, face-to-face, and mobile phone discussions. The identification and selection of a farmer in any rural area depended on his availability via phone, access to the WhatsApp app, and physical presence in some cases. Table 2 presents the number of farmers that participated in the survey by sub-region and country. A link to the survey was sent via email and WhatsApp to all interviewers to collect the data. Some respondents were sent the link to their WhatsApp, and clicking on the link directed them to Google document with survey questions. Other respondents were asked questions through mobile phone interviews. English, Sesotho, and French versions of the questions were available depending on each country's national language. In some circumstances, face-to-face interviews were done in strict adherence to COVID-19 protocols. The French and Sesotho questionnaire results were analyzed in these respective languages and later translated into English to facilitate the paper write-up.

**Table 2:** Numbers of bean farmers that participated in the survey.

Sub-region	Country	Number of farmers	Total
Eastern Africa	Burundi	17	
	Kenya	40	
	Madagascar	52	
	Tanzania	16	
	Uganda	19	144
Southern Africa	Eswatini	34	
	Lesotho	73	
	Zambia	10	
	Zimbabwe	9	126
Central Africa	Cameroon	6	6
West Africa	Burkina Faso	15	15
	**Total**		**291**

The collected data were analyzed descriptively using exploratory data analysis techniques. The proportion of responses to the survey questions was calculated in Excel and presented as either bar plots or tables. Pie charts were also used to present the proportions of the responses. The presentations of results are done by sub-regions.

## 4 Results and discussion

### 4.1 Eastern Africa

#### 4.1.1 Bean production challenges

**Fig. 2:**
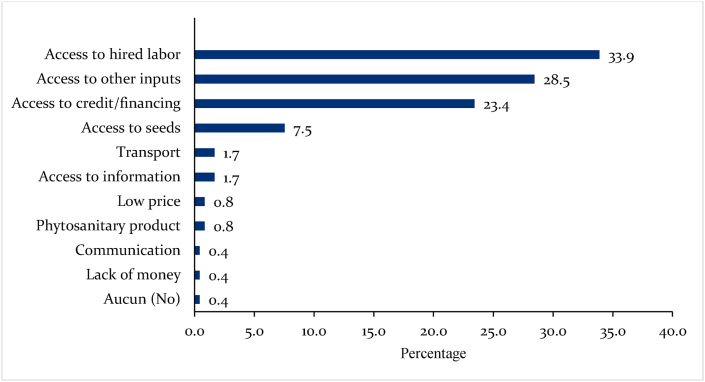
Proportions of responses to the effect of COVID-19 and containment measures on bean production in Eastern Africa.

The challenges faced by bean farmers in Eastern Africa during the pandemic are presented in Fig. 2. The 33.9 percent access to high labor can be attributed to restrictions, fear of getting the disease, high cost of public transportation, and social distancing, all measures implemented by the different governments to curb the pandemic. Second, 29 percent and 23 percent of the farmers mentioned that they faced difficulties accessing agricultural finance and farm inputs, respectively. Other challenges experienced during the pandemic included access to seed and extension information.

Table 2 shows that the pandemic-related challenges to bean production in Eastern Africa varied by country. While the highest proportion of surveyed farmers in Kenya (44 percent) and Burundi (33.3 percent) mentioned that they faced challenges in accessing agricultural credit, 36 percent in Madagascar, half in Uganda, and 57 percent in Tanzania reported difficulties in accessing farm inputs and labor for bean production respectively. Besides, farmers in Uganda indicated that hired labor was scarce (41 percent), and access to agricultural credit was difficult (6 percent). In Tanzania, only 4 percent of the surveyed farmers indicated that they experienced difficulties in accessing credit as shown in Table 3.

**Table 3:** Difficulties experienced by bean farmers during COVID-19 in Eastern African countries.

	Kenya	Burundi	Madagascar	Tanzania	Uganda
Access to hired labor	27.8	29.2	33.7	57.1	40.6
Access to other inputs	8.3	12.5	36.0	7.1	50.0
Access to credit/finance	44.4	33.3	10.5	7.1	6.3
Access to seed	5.6	20.8	9.3	21.4	
Access to market	2.8				
Access to information	11.1				
Access to health services					3.1
Climate					
Transportation difficulties			3.5	7.1	
Low prices			2.3		
Phytosanitary			2.3		
Lack of money			1.2		
Communication			1.2		

Another salient finding is that bean farmers in Kenya, Tanzania, and Madagascar experienced more difficulties than farmers in other countries. For instance, farmers in Kenya experienced challenges in accessing hired labor (27 percent), information (11 percent), and other production inputs (8 percent). Challenges in getting hired labor can be attributed to fear of getting the disease and population following strictly the social distancing government measure to reduce the spread of the pandemic. Also, most farmers in Kenya had already planted their crops but needed to weed them and spray pesticides later Nchanji et al., 2021a, Nchanji et al., 2021b). They also reported seed unavailability (6 percent) and difficulties accessing the market (3 percent).

On the other hand, despite less restrictive containment measures in Tanzania and Burundi, nearly a-fifth of bean farmers in both countries reported that they encountered difficulties in accessing seed. Equal proportions (7 percent) of the surveyed farmers in Tanzania also reported that they experienced challenges accessing agricultural credit. Additionally, Tanzanian farmers said they experienced transportation difficulties. In Burundi, labor shortages (29 percent) and access to other inputs (13 percent) were the most identified bean production difficulties associated with the pandemic.

#### 4.1.2 Food security

While the pandemic's impact on all dimensions of food security cannot be quantified in the long-term, short, and mid-term can be projected based on the immediate impacts of COVID-19 on food systems. The immediate impact of COVID-19 on bean production are transmitted downstream to food supply and consumption. Thus, bean producers were asked to state if household food consumption was affected during the pandemic. Farmers were asked to state how the pandemic affected the frequency of food consumption. The pooled results presented in Table 4 show that most households in Eastern Africa ate twice during the pandemic. The results also suggest that Uganda was the most affected country in Eastern Africa, with all surveyed farmers indicating that they ate once per day during the pandemic.

**Table 4:** Proportions of responses to the effect of the pandemic on the frequency of food consumption in Eastern Africa.

	Kenya	Burundi	Madagascar	Uganda	Pooled
Once		15.4	4.17	100.0	19.2
Twice	80.0	61.5	91.67		61.5
Thrice	10				3.8
Four times and more					
Food shortage	10.0		4.17		15.4

On the other hand, 96 percent, 80 percent, and nearly 62 percent of farmers in Madagascar, Kenya, and Burundi reported that they ate twice per day during the pandemic, respectively. A tenth of the farmers in Kenya indicated they experienced food shortages, which suggests that food access and availability were negatively impacted by governments' restrictive measures to combat the spread of the disease. None of the respondents in Tanzania reported that the pandemic had caused changes in the number of times they ate during the pandemic.

**Fig. 3:**
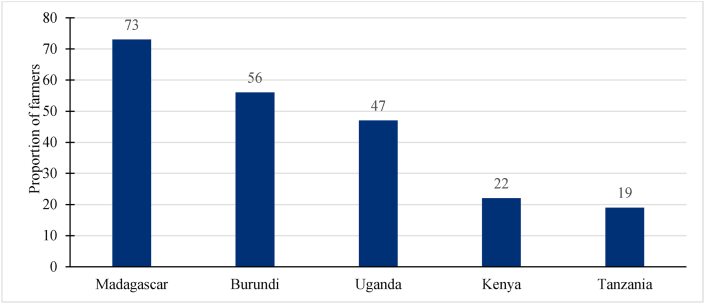
Impact of COVID-19 pandemic on food consumption patterns in Eastern Africa.

Fig. 3 displays the proportions of farmers' responses to the pandemic's impact on food consumption patterns. More farmers in Madagascar (73 percent), Burundi (56 percent), and Uganda (47 percent) than those in Kenya (22 percent) and Tanzania (19 percent) indicated that their food consumption patterns changed during the pandemic. While a less severe impact of the pandemic on food consumption patterns in Tanzania was expected, the higher proportion of farmers in Burundi was unexpected since the two countries' responses to the pandemic were not as restrictive as those in Kenya and Uganda. A possible explanation for changes in Burundi's food consumption patterns could be its dependency on intra-Africa food imports (Tralac, 2020). Most EAC and COMESA imposed strict COVID-19 containment measures, possibly impacting Burundi's food imports.

The immediate impact of COVID-19 and governments' containment measures on the food system are transmitted in myriad ways. Farmers who affirmed that the pandemic affected food consumption patterns identified several pathways for COVID-19 shocks on food consumption. In Kenya and Uganda, the pandemic and containment protocol caused food shortages. This could be attributed to production and distribution difficulties experienced during the pandemic. Second, with millions of jobs lost due to unprecedented disruption of economic activities (Bavier and Paravicini, 2020; World Bank, 2019), livelihoods and food consumption were not spared either (Fox and Signé, 2020). About 36 percent, 20 percent, and 3 percent of farmers in Burundi, Uganda, and Kenya lost income during the pandemic, causing changes in food consumption patterns, respectively (Table 5). The low-income loss in Kenya can be attributed to the activity farmers were carrying out when COVID struck. In Kenya, crops were already planted, and farmers were relying on maize and other crops stored from the previous season for food and to generate income, while in Uganda and Burundi, planting was ongoing, and so more money was spent to get inputs and seed, food as prices and cost of transport increased. Additionally, farmers in Burundi and Tanzania indicated that the pandemic's effect on food consumption patterns triggered changes in food and diet quality and reduced quantities and frequency of food consumption. In Madagascar, disruptions in food consumption patterns were also caused by changes in food and diet quality and reductions in quantity and frequency of consumption, and income losses.

**Table 5:** Proportions of pathways for transmission of the impact of COVID-19 on food security in Eastern Africa.

	Kenya	Burundi	Madagascar	Tanzania	Uganda
Food shortage	90.32				70
No income/lack of money	3.23	36.4	6.1		20
Increased consumption	6.45				10
Increased commodity prices					
Change of diet and quality		27.3	30.3	40	
Reduction in quantity and frequencies		36.4	36.4	60	
Hygiene			27.3		

### 4.2 Southern Africa

#### 4.2.1 Bean production challenges

**Fig. 4:**
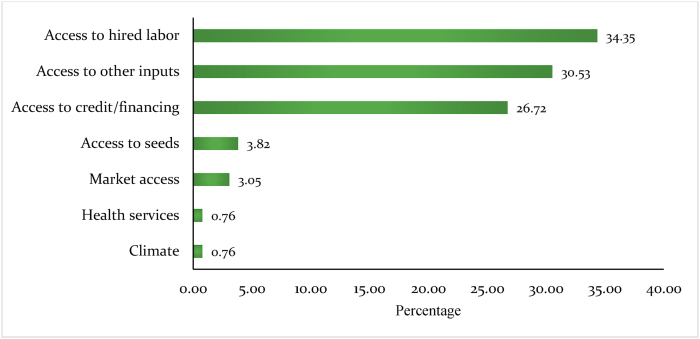
Proportions of difficulties experienced by bean farmers in Southern Africa during the COVID-19 pandemic.

Access to farmworkers and farm inputs were the main consequences of coronavirus and government restrictions in Southern Africa. About 27 percent of the farmers identified the inability to access agricultural financing as another impact of the pandemic on bean production (Fig. 4). Other challenges across the Southern Africa sub-region was access to seed and output markets.

At country levels (Table 6), access to input market challenge was more pronounced in Eswatini and Zimbabwe. Access to hired labor was problematic in Lesotho, Zambia, and Eswatini. About forty-two percent of farmers in Eswatini and 28 percent in Lesotho reported low access to agricultural finance as one of the pandemic-related bean production challenges. The result is in tandem with Maher's and Baskaran's (2020) observation that increasing smallholder farmers' access to credit would quickly reverse the pandemic's impacts on the agricultural sector in Sub-Saharan Africa.

**Table 6:** Challenges experienced by bean farmers during COVID-19 in Southern Africa by country.

	Eswatini	Lesotho	Zambia	Zimbabwe
Access to other inputs	38.0	13.8	16.7	36.4
Access to hired labor	34.2	41.4	41.7	9.1
Access to credit/financing	27.8	27.6	41.7	
Access to seeds		10.3		18.2
Transport				
Market access		3.4		27.3
Phytosanitary product				
Low price				
Lack of money				
Communication				
Climate		3.4		
Access to health services				9.1

#### 4.2.2 Food security

**Fig. 5:**
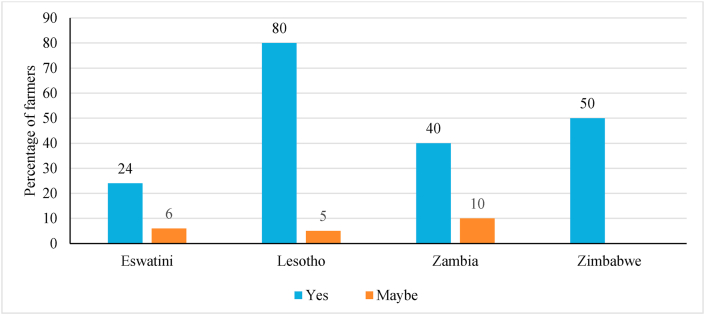
Proportions of responses of the impact of the pandemic on changes in food consumption in Southern Africa.

In Fig. 5, Lesotho was the most affected country in the Southern Africa sub-region in terms of changes in the number of times food was consumed during the pandemic. About 80 percent of the farmers reported changes in the number of times they consumed food. Farmers in Zimbabwe (50%) and Zambia (40%). On the other hand, less than one-quarter - 24 percent of farmers in Eswatini changed the number of times they consumed food during the pandemic (Fig. 7). These differences in proportions could be attributed to varying levels of application of COVID-19 containment measures, which were second and third most affected in terms of the number of times they ate food per day, respectively.

**Fig. 6:**
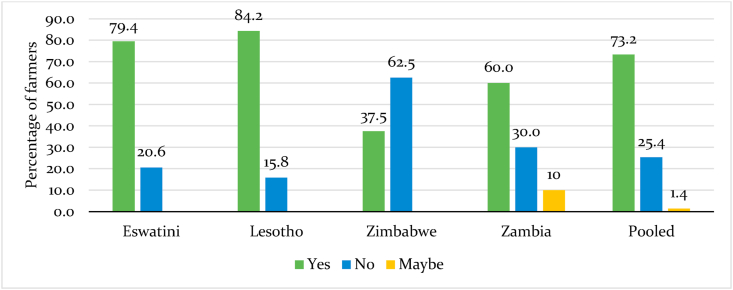
Impact of COVID-19 pandemic on food consumption patterns in Southern Africa.

Furthermore, 73 percent of Southern Africa farmers reported that COVID-19 caused changes in food consumption patterns (Fig. 6). Comparison of the results shows that farmers in Lesotho and Eswatini were the most affected. In contrast, Zimbabwean farmers reported the least changes in consumption patterns (Fig. 6).

**Fig. 7:**
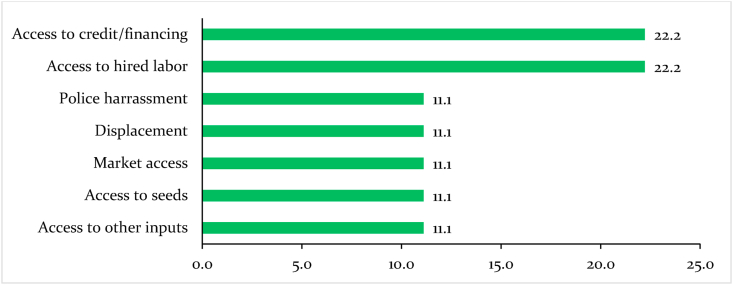
Proportions of difficulties experienced by bean farmers in Cameroon during the COVID-19 pandemic.

Critical pathways for the transmission of the impacts of the pandemic on bean production to consumption were food shortages in Eswatini (96%) and Lesotho (77%). Half of the farmers in Zambia and three-quarters of those in Zimbabwe also reported food shortages during the pandemic (Table 7). Besides, another half of Zambia farmers cited low income, increased household food consumption, and increased food prices as causes of changes in food consumption patterns. On the other hand, one-third of farmers in Zimbabwe identified loss of income as another driver of changes in food consumption during the pandemic (Table 7). These findings underscore the proposition of the entitlement approach to food security. The pandemic possibly impacted production-based, own-labor, as well as market-based and remittance based sources of food in Southern Africa. Loss of both farm and off-farm incomes due to the pandemic's direct and indirect effects could have reduced farmers' purchasing power, thereby undermining food consumption. Additionally, schools' closure undermined households' efforts to improve food security situations due to the many mouths they have to feed.

**Table 7:** Pathways of transmission of the impact of COVID-19 on food security in Southern Africa.

	Eswatini	Lesotho	Zambia	Zimbabwe
Food shortage	96.3	76.9	50	66.7
No income/lack of money	3.7	7.7	16.7	33.3
Increased consumption		15.4	16.7	0.0
Increased commodity prices			16.7	0.0
Change of diet and quality				
Decreased quantity/consumption frequency				
Hygiene				

### 4.3 Central Africa

#### 4.3.1 Bean production

The data were only collected in Cameroon and, therefore, used to show possible short-term effects of the pandemic on bean farming and food security in the Central African sub-region. Equal proportions or 44 percent of farmers in Cameroon identified challenges in accessing credit and hired labor as the immediate effects of the pandemic on bean production (Fig. 7). Remittances from family members and relatives in urban centers under stricter enforcement of containment measures could have caused farmers' liquidity challenges to finance agricultural production. Another 33 percent of the farmers reported that they experienced challenges accessing inputs, seed, and the output markets (Fig. 7). The low proportions of farmers who reported access to farm inputs and seed could be because the survey and the beginning of COVID-19 restrictions coincided with the second season of Cameroon food production calendar's (bean is grown only in the West region.

### 4.4 Food security

**Fig. 8:**
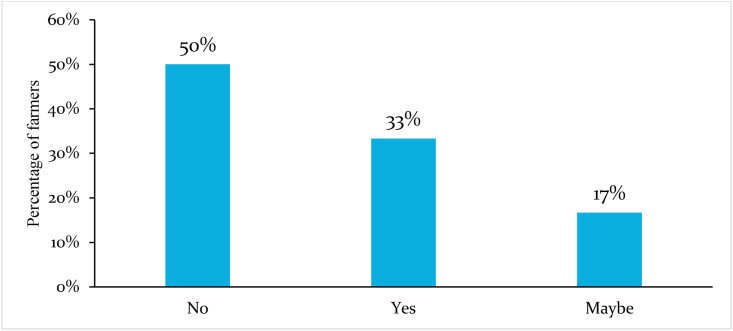
Proportions of difficulties experienced by bean farmers in Central Africa during the COVID-19 pandemic.

Nearly 33 percent of the farmers affirmed that their food consumption had changed during the pandemic, while 17 percent were not sure (Fig. 8). Two-thirds of those who reported changes in food consumption indicated that their households had been affected by food shortages. (Fig. 8). Another one-third reported that they were eating twice a day. However, these results are interpreted with much caution because they are not representative of bean farming households in Cameroon's survey area because of the small sample size.

### 4.5 Western africa

**Fig. 9:**
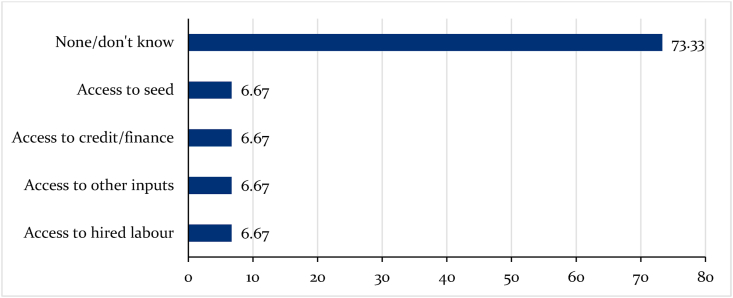
Proportions of difficulties experienced by bean farmers in Burkina Faso during the COVID-19 pandemic.

The Sahel countries, which are predominantly western African countries, faced numerous challenges pre-COVID-19. The challenges include insecurity, political instability, social conflicts, and climate change. For instance, FAO (2020) projects that 17 million people in West Africa will be acutely food insecure following the implementation of restriction measures to contain the virus. However, due to data limitations, we used data collected from farmers in Burkina Faso to portray the region's food security situation during the pandemic. Most or 73 percent of farmers in Burkina Faso at the time of the survey reported that they had not experienced any bean production challenges related to COVID-19 (Fig. 9). This could be attributed to some not being unable to identify any immediate impact because of the survey's timing. Additi0nally, the cropping season had hardly begun when the pandemic stroke most bean production areas in Burkina Faso. Even so, about 28 percent of them reported challenges related to access to seed, credit, farm inputs, and hired labor as the immediate impacts of the pandemic on bean production in irrigated areas.

Notwithstanding farmers responses in Fig. 9, existing agricultural production vulnerabilities like low use of input fertilizer and improved seed, climate change, and pests and diseases, as well as insecurity and poverty, are likely to exacerbate the implication of COVID-19 on food security in Burkina Faso and the sub-region at large (FAO, 2020). Recent statistics show that three million people in Burkina Faso are already facing starvation due to COVID-19. Additionally, the country's social and political instability could also fuel the population's vulnerability to food insecurity during the pandemic. These assertions are affirmed by about forty percent of the farmers who indicated that they were already grappling with food security problems due to the pandemic.

## 5 Potential effects of COVID-19 on Sustainable Development Goals

The pandemic has exposed the vulnerabilities of food systems in developing countries. Concerns about the immediate impacts of COVID-19 on Sub-Saharan Africa have turned into fears and raised uncertainty over the progress in achieving SDGs 1 and 2. The initial results suggest economies in the region may find themselves in unprecedented situations where the pandemic threatens to undo years of progress in ending hunger and poverty. The UN identifies developments in the agricultural sector as central to ending poverty and hunger in the region. However, its contribution to the SDGs may be disrupted because of the coronavirus related effects. For instance, results presented herein suggest reduced access to financial services across the sub-region. This could be attributed to the diversion of agricultural financial support to health services, which threatens investment in agricultural development programs such as technology acquisition, mechanization, research, and infrastructure. Additionally, the unprecedented macroeconomic effects of COVID-19 have reduced private transfers to agriculture, reducing farmers' liquidity. Taken together, reduced financing of the agricultural sector may undermine access to technologies such as improved seed, fertilizer, and irrigation, which could lead to a decline in crop yields, food shortages, and hunger.

Given the nature of government restrictions during the pandemic, agricultural productivity is likely to slow down because of production-related constraints caused by the pandemic. The result indicates that the internal containment measures have disrupted access to seed and farm inputs. This is likely to undermine the utilization of improved agricultural technologies, which was already low across the sub-regions. The region is also highly dependent on international trade for the supply of most farm-input; therefore, lockdowns and cross-border restrictions are likely to worsen inputs' availability, which would cause a decrease in the production of food staples. This would have short-term and long-term implications for the agricultural sector's ability to reduce the number of hungry and malnourished people in the region. For instance, poor farming households may lose access to their own produced food due to low agricultural production of food staples such as cereals and legumes. Furthermore, border closures may limit cross-border trade of important food commodities, which would exacerbate the region's already dire food security situation. Additionally, poor access to the output market may reduce farm income, which, in turn, would lead to low economic access to food and slow progress from poverty.

## 6 Response to COVID-19 pandemic across regions

Although many governments in Sub-Saharan Africa are easing their initial responses to the outbreak of COVID-19 to avert dire social and economic impacts of the pandemic, essential restrictions remain in place, and the immediate consequences of the containment measures will still be felt in the near future. Thus, anticipatory actions have been taken by both government and private players to avert or reduce the impacts of the pandemic on food security. In Burkina Faso, the government's support to the agricultural sector during the pandemic included a $55,891,500 input subsidy program, food assistance to households, and local businesses' support (Nwafor, 2020). Additionally, the Burkina government bought stocks of consumer products and strengthened food prices surveillance to reduce the pandemic's impacts on food security. The Kenyan government enhanced and expanded smallholder farmers' access to credit signature schemes.

The government in the region has also expanded mobile-based payments and credit to enhance access to agricultural financing. The Zambian central bank increased the limits of the daily transaction for small-scale farmers and businesses. Central governments in Kenya, Uganda, and Tanzania eliminated mobile money charges and increased daily transaction limits. This was to reduce the cost of doing business and ensure the liquidity of smallholder farmers. Additionally, most countries across the region offered income and VAT tax reliefs.

Furthermore, the Ugandan government supported scaling up digital information sharing models as well as digital extension services. In partnership with the Alliance of Green Revolution in Africa (AGRA), the Ugandan government strengthened existing market linkages. Other measures initiated across sub-regions were increased public-private partnerships in agricultural programs to sustain food production, food distribution channels open, and agribusiness. For instance, agro-chemical and agri-entrepreneurs in Kenya, with the government's support, facilitate farmers' access to extension services and information.

The bean value chain has also received attention from international organizations. For instance, the Pan-Africa Bean Research Alliance (PABRA) is providing digital agronomic trainings and information through programs facilitated collaboratively with government extension workers. PABRA has implemented projects across the region that offer mechanization solutions to labor shortage through the provision of planters and threshers and mechanization training. Furthermore, PABRA is involved in the off-season production of seed in collaboration with the private sector and National Agricultural Research Systems (NARS) to meet the demand for seed in upcoming seasons. PABRA is also working in Eastern, Southern, Central, and West Africa to strengthen bean production, distribution, and consumption amid the pandemic. For instance, PABRA collaborates with producer organizations, faith-based organizations, and community-based organizations to distribute bean seed, offer farmer training, encourage aggregation and group marketing and bean processing to scale up the commercialization of innovative bean products. To overcome the credit constraints reported by farmers, the alliance is co-financing women and youth businesses, especially acquiring needed infrastructure for business continuity. These support programs are intended to strengthen the bean value chain's resilience against the effects of COVID-19.

## 7 Conclusion and policy implications

The global spread of coronavirus and the unprecedented containment measures threaten countries in Sub-Saharan Africa's ability to eliminate hunger and poverty. The pathways of the impact of the pandemic on food security begin at the farm level. Farm-level impacts are expected to be transmitted across agri-food chains, such as market access, logistics, and food processing due to disruptions caused by transport, lockdowns, and cross-border restrictions. The end-point is household impacts, including loss of income, limited or unstable availability and access to food, food insecurity, and poverty. These would exacerbate the already dire food security and poverty situations in Sub-Saharan Africa. It would also reverse the gain made in eliminating hunger and poverty.

Based on the immediate impacts of COVID-19 identified by this study, we recommend a package of policy interventions to revitalize and build farmer's resilience against the pandemic's devastating effects. At national levels, governments should monitor the consequences of the pandemic on agriculture, food systems, and incomes and regularly use the information to formulate or update existing responses or policies. Therefore, the paper implores governments across the region to strengthen the food systems' resilience against present and future shocks. This calls for an immediate transformation of food systems in all the sub-regions.

## Funding

We acknowledge technical support from the Pan-African Beans Research Alliance (PABRA) through the Alliance of Bioversity International and the International Center for Tropical Agriculture (CIAT), and financial support from Global Affairs Canada and the Swiss Agency for Development and Cooperation (SDC) to undertake this study. We also thank the 10.13039/100000865Bill and Melinda Gates Foundation for funding this paper's publication under the “Accelerated varietal improvement and seed delivery of legumes and cereals in Africa” project. Many thanks go to farmers and other stakeholders who provided information without which this study would not have been successful.

## Declaration of competing interest

There is no conflict of interest.
